# Deployment-Oriented Evaluation of Lightweight IMU-Based Human Activity Recognition: On-Device Efficiency and Streaming Feasibility

**DOI:** 10.3390/s26175678

**Published:** 2026-09-07

**Authors:** Inho Gil, Seongmin Ha, Jihwan Oh, Donggyu Lee, Soonwoong Hwang, Joonkyu No, Wansoo Kim

**Affiliations:** 1 Department of Robotics, Hanyang University, 222 Wangsimni-ro, Seongdong-gu, Seoul 04763, Republic of Korea; inho7685@hanyang.ac.kr (I.G.); iittyy10@hanyang.ac.kr (J.O.); dong2391@hanyang.ac.kr (D.L.); 2 Department of Mechatronics Engineering, Hanyang University, 222 Wangsimni-ro, Seongdong-gu, Seoul 04763, Republic of Korea; gktjdals9416@hanyang.ac.kr; 3 Department of Robotics, Hanyang University ERICA, 55 Hanyangdaehak-ro, Sangnok-gu, Ansan 15588, Republic of Korea; hswfile@hanyang.ac.kr (S.H.); njkonly@hanyang.ac.kr (J.N.)

**Keywords:** inertial measurement units, human activity recognition, wearable sensors, deep learning models, lightweight, on-device efficiency, transformer, TCN, LSTM

## Abstract

On-device Human Activity Recognition (HAR) requires balancing accuracy and deployment efficiency on constrained hardware. We present Lightweight Human Activity Recognition (L-HAR), a controlled comparison of Baseline and Lightweight Temporal Convolutional Network (TCN), Transformer, and Long Short-Term Memory (LSTM) models using data sampled at 100Hz from three inertial measurement units (IMUs) worn by one participant. Evaluations covered classification, model footprint, multiply–accumulate operations (MACs), offline CPU latency, Desktop/Raspberry Pi streaming, 8-bit integer (INT8) quantization, and software-estimated Raspberry Pi energy efficiency. Lightweighting reduced parameters by up to 95.5%, model size by 94.0%, and MACs by 87.6–95.5%, with accuracy reductions of 0.8–2.2 percentage points (pp) and sub-millisecond offline latency for all Lightweight models. Baseline and Lightweight models sustained approximately 100Hz message/inference rates on Desktop, whereas no Raspberry Pi configuration reached 100Hz inference. Among Lightweight models, Transformer achieved 9.381ms End-to-End (E2E) latency and 66.925Hz inference. INT8 quantization changed accuracy and F1-score by less than 0.13 pp; Quantized TCN achieved the best Raspberry Pi streaming result (9.066ms E2E; 69.928Hz) with an estimated 49.933mJ per inference. These results demonstrate architecture- and backend-dependent deployment behavior and the need for direct target-platform evaluation.

## 1. Introduction

Human Activity Recognition (HAR) refers to the automatic identification of human activities from sensor signals, including the classification, segmentation, and detection of simple and complex human behaviors [1]. HAR covers static postures, locomotor activities, and Activities of Daily Living (ADLs), supporting applications in healthcare monitoring, rehabilitation, safety assessment, and activity-aware assistance [2,3]. Sensors used for HAR can be broadly categorized according to how information about motion and activity is acquired. Environmental sensors, including RGB or depth cameras and LiDAR, observe human activities from the surrounding environment, whereas body-worn sensors, such as Inertial Measurement Units (IMUs), pressure sensors, and physiological sensors, directly measure motion or physical responses from the wearer [4]. Because each sensing modality captures different aspects of human activity and imposes different deployment requirements, sensor selection is a fundamental design consideration in practical HAR systems.

Vision-based HAR captures rich spatial information on human posture, movement, and environmental interactions, but remains sensitive to camera configuration, illumination, occlusion, and environmental changes. Visual data also raise privacy concerns [5], and continuous video acquisition and analysis impose substantial bandwidth, storage, and computational demands, potentially increasing latency and power consumption in resource-constrained systems [6]. In contrast, body-worn IMUs directly capture the wearer’s motion without external infrastructure or continuous visual observation, and their compact size, relatively low cost, low power consumption, and independence from lighting and line-of-sight conditions make them suitable for mobile and on-device HAR. Accelerometer and gyroscope signals further provide multivariate temporal information well suited to sequence-based models, motivating the use of body-worn IMUs in this study [7].

Despite these advantages, IMU-based HAR remains sensitive to sensor placement and orientation, individual movement patterns, and sensor noise. Moreover, limited environmental context can hinder the recognition of activities with similar motion patterns. Accordingly, previous IMU-based HAR studies have extensively investigated signal robustness [8], data heterogeneity [9], and generalization across users, devices, and sensor placements [10]. Health-oriented HAR studies have also identified device placement, missing data, participant diversity, and validation under free-living conditions as persistent methodological challenges [11].

Beyond these recognition-oriented challenges, practical IMU-based HAR must account for on-device computational constraints. Although multichannel inertial signals provide informative temporal patterns, processing them with deep learning models can impose substantial computational cost and energy consumption on resource-constrained hardware [12]. In response, recent studies have demonstrated real-time sensor-embedded HAR on low-cost microcontrollers [13] and investigated model-compression techniques, such as pruning and quantization, to reduce model size and computational cost [14]. Knowledge-distillation-based lightweight HAR has also been investigated for resource-constrained deployment [15]. Real-time deployment has also been demonstrated in wearable exoskeleton systems using IMU and encoder signals on an edge device [16].

Collectively, these studies address complementary aspects of embedded HAR through specific model families, compression strategies, or application-specific deployment settings. Because their sensing configurations, model architectures or compression strategies, hardware platforms, and runtime settings differ, direct comparisons across studies are not always straightforward, which is consistent with the broader need for representative and reproducible inference benchmarking across heterogeneous systems [17]. Moreover, reductions in parameters or theoretical computational complexity do not necessarily lead to proportional improvements in wall-clock latency or real-time streaming performance. Offline inference latency may not fully reflect system-level effects during continuous streaming, such as message age, processing backlog, and discrepancies between the sensor input rate and actual inference throughput. These considerations motivate a unified evaluation of classification performance, model footprint, computational complexity, offline latency, online streaming feasibility, and energy efficiency across multiple model architectures and target platforms.

**Figure 1:**
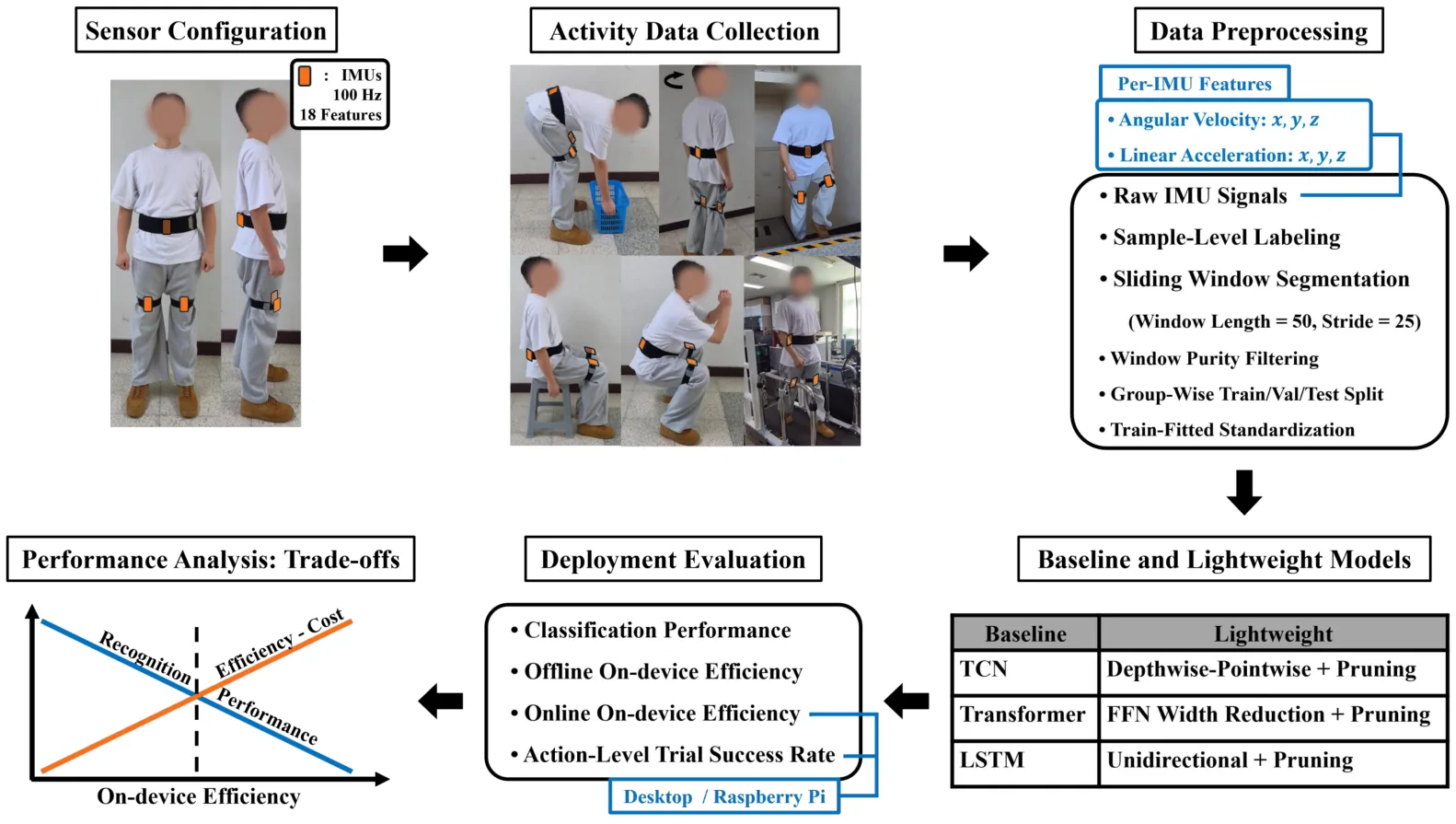
Overview of the proposed Lightweight Human Activity Recognition (L-HAR) framework, comprising the three-IMU sensor configuration, activity data collection, data preprocessing, Baseline and Lightweight models, deployment evaluation, and performance–efficiency trade-off analysis.

To address this need, we present Lightweight Human Activity Recognition (L-HAR), a controlled, deployment-oriented framework for comparing Baseline and Lightweight variants of three representative sequence models—Temporal Convolutional Network (TCN), Transformer, and Long Short-Term Memory (LSTM)—under a unified three-IMU data collection, signal preprocessing, model comparison, and deployment-evaluation pipeline (Figure 1). L-HAR comprehensively evaluates classification performance, model footprint, model-level computational complexity, offline Core/Inference latency on Desktop, online streaming behavior on Desktop and Raspberry Pi platforms, and software-estimated energy efficiency during Raspberry Pi streaming. This unified framework enables architecture- and platform-dependent trade-offs between recognition performance and on-device efficiency to be assessed under consistent experimental and runtime conditions. Because the dataset was collected from a single participant, the study focuses on the effects of model architecture, lightweighting strategy, and target platform on deployment performance rather than population-level generalization. The sequence architectures and lightweighting techniques employed in this study are established methods. Accordingly, the contribution of L-HAR lies in the controlled, deployment-oriented evaluation of TCN, Transformer, and LSTM under common sensing, input, preprocessing, initial-training, and evaluation conditions, and in linking offline model-level efficiency with target-platform streaming behavior and software-estimated energy efficiency. This controlled evaluation enables architecture-dependent relationships between model-level efficiency and target-platform performance to be examined under consistent experimental and runtime conditions.

Our contributions are threefold: (1) We present L-HAR, a unified deployment-oriented evaluation framework that jointly quantifies classification performance, model-level computational complexity, offline on-device efficiency (parameters, on-disk FP32 model size, and Core/Inference latency measured on Desktop), and online on-device efficiency (End-to-End (E2E)/Pipeline/Core latency and message/inference rates measured on Desktop and Raspberry Pi, together with software-estimated energy efficiency during Raspberry Pi streaming). (2) We implement and compare Baseline and Lightweight variants of TCN, Transformer, and LSTM under common sensing, input, preprocessing, initial-training, and evaluation conditions, using model-specific lightweighting procedures. (3) Through combined offline and online evaluations, we show that lightweighting can achieve sub-millisecond CPU inference and improve embedded streaming feasibility, while revealing architecture- and platform-dependent differences in streaming behavior, quantization effects, energy efficiency, and action-level trial success rates; these results demonstrate the need for direct target-platform evaluation beyond isolated offline measurements.

The remainder of this paper is organized as follows. Section 2 presents the L-HAR framework and model architectures, while Section 3 describes the data collection, preprocessing, training, and evaluation setup. Section 4 reports the classification performance, on-device efficiency, computational complexity, software-estimated energy efficiency, class-level recognition, and action-level trial results; Section 5 discusses the resulting performance–efficiency trade-offs, deployment implications, and limitations; and Section 6 concludes the paper.

## 2. Methodology

### 2.1. Framework Overview

The proposed L-HAR framework provides a unified pipeline for evaluating the deployment-oriented behavior of lightweight IMU-based HAR models under consistent sensing, preprocessing, training, and runtime conditions (Figure 1). Rather than proposing new recognition algorithms, the framework applies established lightweighting techniques to three representative sequence models (TCN, Transformer, and LSTM) and characterizes their model footprint, model-level computational complexity, and offline CPU efficiency on Desktop, as well as their online streaming behavior on Desktop and Raspberry Pi. The Lightweight models were additionally subjected to architecture-specific 8-bit integer (INT8) quantization for Raspberry Pi evaluation, and software-estimated energy efficiency was profiled for the Baseline, Lightweight, and Quantized configurations on Raspberry Pi. All Baseline and Lightweight variants share the same three-IMU input, preprocessing, and training pipeline, enabling architecture- and platform-dependent trade-offs to be compared under controlled conditions in this single-participant study. The framework retains common evaluation criteria while allowing model-specific lightweighting and platform-specific deployment, enabling extension to additional architectures and target platforms.

Section 2.2, Section 2.3, Section 2.4 and Section 2.5 describe the model architectures, quantization, input representation, and computational-complexity estimation; Section 3 details the experimental setup.

### 2.2. Model Architectures and Lightweight Variants

**Figure 2:**
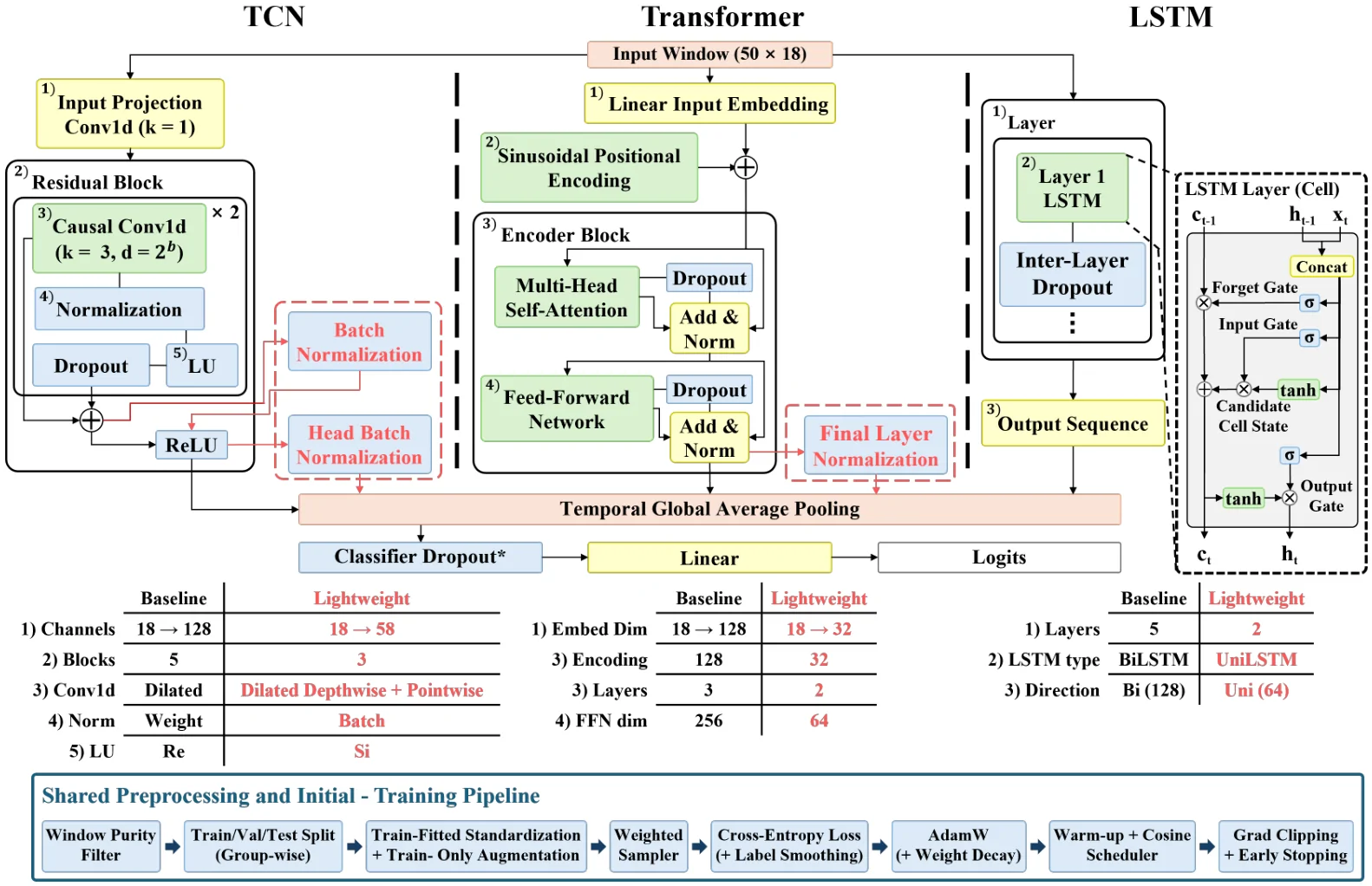
Detailed comparison of the Baseline and final Lightweight TCN, Transformer, and LSTM architectures used in L-HAR. The displayed Lightweight configurations correspond to the final models used in the evaluation after the model-specific pruning procedures described in Section 2.2. Black elements and values denote components used in the Baseline configuration or shared by both variants, whereas red elements and values indicate Lightweight-specific replacements, additions, or final values that differ from the Baseline configuration. The classifier Dropout marked with an asterisk is applied to all displayed configurations except for the final Lightweight TCN, which applies Head BatchNorm before temporal global-average pooling and passes the pooled features directly to the Linear classifier. The figure also shows the common 50×18 input representation, temporal global-average pooling, model-specific classification-head components, the LSTM-cell operation, and the shared preprocessing and initial-training pipeline.

Figure 2 presents the Baseline and final Lightweight architectures of the TCN, Transformer, and LSTM models used in the evaluation. The final Lightweight configurations correspond to the models obtained after the model-specific pruning procedures described below, whereas the intermediate initial Lightweight configurations are described in the text to clarify the lightweighting process. The figure also shows their common 50×18 input representation, model-specific processing blocks, temporal global-average pooling, model-specific normalization and classification-head components, the LSTM-cell operation, and the shared preprocessing and initial-training pipeline.

We used three sequence models: TCN (1D dilated convolutions for long-range dependencies with high parallelism) [18], Transformer (self-attention capturing global context) [19], and LSTM (gated recurrent neural network mitigating vanishing gradients) [20]. For each architecture, we evaluated a Baseline model and a Lightweight variant designed to improve on-device efficiency. Each Lightweight model was first trained using a reduced initial architecture and was subsequently subjected to model-specific structured pruning [21]. The model-specific lightweighting techniques were as follows:

- **TCN**: The Baseline TCN consisted of five residual blocks with 128 channels. The initial Lightweight TCN used five residual blocks with 64 channels and replaced the standard convolutions with depthwise–pointwise separable convolutions [22], implemented as causal 1D convolutions with batch normalization (BatchNorm) and sigmoid linear unit (SiLU) activation. For both TCN variants, the convolutional kernel size was fixed at k=3, and the dilation factor of block *b* followed $d_{b}=2^{b}$, where b=0,…,B−1 for a model with *B* residual blocks. The Lightweight TCN retained this dilation schedule. After initial training, residual-block pruning evaluated progressively shallower architectures down to three blocks, with the final depth in each run selected based on validation performance. Structured channel pruning was then performed using the aggregated absolute BatchNorm scaling factors as channel-importance scores, following the channel-selection principle of network slimming [23]. A pruning ratio of 0.10 reduced the channel width from 64 to 58. The final Lightweight TCN additionally applied Head BatchNorm before temporal global-average pooling, and the pooled features were passed directly to the Linear classifier without the post-pooling classifier Dropout used in the other Baseline and Lightweight configurations.
- **Transformer**: The Baseline Transformer consisted of three encoder layers with an embedding dimension of 128, four attention heads, and a feed-forward network (FFN) dimension of 256. The initial Lightweight Transformer used two encoder layers with an embedding dimension of 32, four attention heads, and an FFN dimension of 128. Because FFN layers constitute a substantial portion of the parameters in Transformer language models [24], the FFN hidden units were subsequently pruned according to their weight-norm importance, while a minimum of 64 units was retained. The Lightweight Transformer therefore consisted of two encoder layers with an embedding dimension of 32 and an FFN dimension of 64. The final Lightweight Transformer additionally applied LayerNorm after the encoder stack before temporal global-average pooling.
- **LSTM**: Bidirectional LSTM architectures have previously been applied to IMU-based HAR to capture forward and backward temporal dependencies [25]. The Baseline LSTM consisted of a five-layer bidirectional LSTM with a hidden dimension of 64 per direction. The initial Lightweight LSTM used a three-layer unidirectional architecture with the same hidden dimension. A two-layer model was subsequently evaluated through structured layer pruning, with the final recurrent depth in each run selected based on validation performance. These modifications reduced recurrent computation, parameters, and memory overhead.

During the pruning procedures, each candidate or progressively pruned model was briefly fine-tuned to mitigate potential recognition-performance degradation. Accordingly, the reported Lightweight results reflect the combined effects of the reduced initial architecture and subsequent structured pruning, rather than the isolated effect of pruning alone. The training and fine-tuning protocol is described in Section 3.4.

### 2.3. Post-Training INT8 Quantization

Post-training INT8 quantization (PTQ) was applied to the Lightweight TCN, Transformer, and LSTM models. The model architectures and the common 50×18 input representation were retained without modification, while the quantization procedure was selected according to the operator characteristics and runtime support of each architecture. The model-specific quantization procedures were as follows:

- **TCN**: Static INT8 weight-and-activation quantization (W8A8) was applied to supported operators of the Lightweight TCN through the PyTorch 2 Export (PT2E) workflow with the XNNPACK quantizer. Calibration was performed using 1024 class-stratified windows sampled exclusively from the training split. The converted model was subsequently lowered to the XNNPACK backend and exported as an ExecuTorch program for Raspberry Pi deployment.
- **Transformer**: Dynamic INT8 PTQ with the QNNPACK backend was applied to the supported Linear layers, including the input embedding, the feed-forward layers in both encoder blocks, and the final classifier. The attention operations, LayerNorm, positional encoding, and temporal global-average pooling remained unquantized.
- **LSTM**: Dynamic INT8 PTQ with the QNNPACK backend was applied to the LSTM module and the final Linear classifier. Unlike the static quantization used for TCN, the dynamic quantization applied to Transformer and LSTM did not require a separate calibration dataset.

The Quantized variants were evaluated on the same group-wise test split as their corresponding Lightweight FP32 source checkpoints to quantify the effect of quantization on classification performance before Raspberry Pi deployment.

### 2.4. IMU Input Representation

The common model input consisted of three-axis angular velocity and three-axis linear acceleration signals from the left thigh, right thigh, and pelvis IMUs. No study-specific sensor fusion or handcrafted orientation-feature extraction was applied. Magnetic-field measurements, orientation estimates, and derived orientation features such as roll, pitch, and yaw were excluded from the model input. Although magnetometer measurements can provide additional heading- and orientation-related information, including the three-axis magnetic-field measurements from all three IMUs would increase the input dimensionality from 18 to 27 channels. We therefore retained the six-axis accelerometer–gyroscope representation, which is commonly used in wearable HAR applications, whereas magnetometer measurements are not always included in HAR analysis [26]. For the directional Turning classes in this study, direction-dependent rotational motion was represented directly by the gyroscope angular-velocity signals. The detailed sensor configuration and data-acquisition procedure are described in Section 3.1.

For each sensor location s∈{LT,RT,P}, the input feature vector at sample index *t* was defined as

(1) $$u_{t}^{\left(s\right)}={\left[\omega_{x,t}^{\left(s\right)},\omega_{y,t}^{\left(s\right)},\omega_{z,t}^{\left(s\right)},a_{x,t}^{\left(s\right)},a_{y,t}^{\left(s\right)},a_{z,t}^{\left(s\right)}\right]}^{T}\in R^{6},$$

where LT, RT, and P denote the IMUs attached to the left thigh, right thigh, and pelvis, respectively. The variables $\omega_{x,t}^{\left(s\right)}$, $\omega_{y,t}^{\left(s\right)}$, and $\omega_{z,t}^{\left(s\right)}$ denote the three-axis angular-velocity measurements, while $a_{x,t}^{\left(s\right)}$, $a_{y,t}^{\left(s\right)}$, and $a_{z,t}^{\left(s\right)}$ denote the corresponding three-axis linear-acceleration measurements. Thus, each IMU contributed six input features.

The within-sensor feature order was fixed as $\left[\omega_{x},\omega_{y},\omega_{z},a_{x},a_{y},a_{z}\right]$. The feature vectors from the three sensors were concatenated in the order left thigh, right thigh, and pelvis as

(2) $$x_{t}=\left[u_{t}^{\left(\mathrm{LT}\right)}∥u_{t}^{\left(\mathrm{RT}\right)}∥u_{t}^{\left(P\right)}\right]\in R^{18},$$

where ∥ denotes concatenation along the feature dimension. More explicitly, the complete feature order was defined as

(3) $$\begin{array}{c} x_{t}=\;[\omega_{x,t}^{\left(\mathrm{LT}\right)},\omega_{y,t}^{\left(\mathrm{LT}\right)},\omega_{z,t}^{\left(\mathrm{LT}\right)},a_{x,t}^{\left(\mathrm{LT}\right)},a_{y,t}^{\left(\mathrm{LT}\right)},a_{z,t}^{\left(\mathrm{LT}\right)},\\ \;\omega_{x,t}^{\left(\mathrm{RT}\right)},\omega_{y,t}^{\left(\mathrm{RT}\right)},\omega_{z,t}^{\left(\mathrm{RT}\right)},a_{x,t}^{\left(\mathrm{RT}\right)},a_{y,t}^{\left(\mathrm{RT}\right)},a_{z,t}^{\left(\mathrm{RT}\right)},\\ \;\omega_{x,t}^{\left(P\right)},\omega_{y,t}^{\left(P\right)},\omega_{z,t}^{\left(P\right)},a_{x,t}^{\left(P\right)},a_{y,t}^{\left(P\right)},a_{z,t}^{\left(P\right)}{]}^{T}\in R^{18}. \end{array}$$

Consequently, each synchronized sample was represented by an 18-dimensional feature vector, and the same sensor and feature ordering was applied to all Baseline and Lightweight model variants. After the preprocessing and window-segmentation procedures described in Section 3.3, these feature vectors formed the common 50×18 model input shown in Figure 2, where the first dimension corresponded to the 50 temporal samples and the second dimension corresponded to the 18 concatenated IMU features.

### 2.5. Computational Complexity Estimation

To characterize the model-level computational workload, multiply–accumulate operations (MACs) per inference were analytically estimated for the Baseline and final Lightweight architectures shown in Figure 2, using a batch size of one and the common 50×18 input defined in Section 2.4. The model-specific MAC calculations were defined according to the corresponding architectures described in Section 2.2.

For TCN, the MAC count of the Baseline architecture was calculated as

(4) $$M_{\mathrm{TCN},B}=TDN+2BTN^{2}K+NC,$$

whereas the MAC count of the Lightweight TCN using depthwise–pointwise separable convolutions was calculated as

(5) $$M_{\mathrm{TCN},L}=TDN+2BT\left(NK+N^{2}\right)+NC.$$

For Transformer, the MAC count was calculated as

(6) $$M_{\mathrm{Tr}}=TDE+L_{\mathrm{Tr}}\left(4TE^{2}+2T^{2}E+2TEF\right)+EC.$$

For LSTM, the MAC count was calculated as

(7) $$M_{\mathrm{LSTM}}=T\sum_{l=1}^{L_{\mathrm{LSTM}}}d\left(4HI_{l}+4H^{2}\right)+dHC,$$

where $I_{1}=D$ and $I_{l}=dH$ for l>1.

Here, *T* denotes the sequence length, *D* the input-feature dimension, and *C* the number of output classes. For TCN, *B*, *N*, and *K* denote the number of residual blocks, channel width, and convolutional kernel size, respectively. For Transformer, $L_{\mathrm{Tr}}$, *E*, and *F* denote the number of encoder layers, embedding dimension, and feed-forward network dimension, respectively. For LSTM, $L_{\mathrm{LSTM}}$, *H*, $I_{l}$, and *d* denote the number of recurrent layers, hidden dimension, input dimension of recurrent layer *l*, and number of recurrent directions, respectively, with d=2 for the Baseline bidirectional LSTM and d=1 for the Lightweight unidirectional LSTM.

The MAC estimates include the dominant convolutional, linear, attention, feed-forward, recurrent, and classification operations but exclude non-MAC operations such as normalization, activation, Softmax, pooling, element-wise gate operations, residual addition, and bias addition. Because post-training INT8 quantization changes numerical precision and backend execution without changing the logical model architecture, each Quantized variant was assigned the same logical MAC count as its corresponding Lightweight model.

## 3. Experimental Setup

### 3.1. Data Collection

Data were collected from a single participant in a controlled experimental setting. Three Xsens MTw Awinda IMUs were used, each equipped with a three-axis accelerometer, gyroscope, and magnetometer. All IMU signals were collected at 100Hz, and sensor data acquisition and synchronization were implemented using ROS 2 Humble on Ubuntu 22.04.

The three IMUs were attached to the pelvis near the L3 region and to the left and right thighs, as illustrated in Figure 1. This placement was selected to capture major lower-body movements relevant to the recognition of fundamental activities and ADLs. Previous research has identified the thigh and pelvis regions as effective sensor locations for recognizing basic human activities [27]. Accordingly, the pelvis and both thighs were selected to balance lower-body motion coverage and wearer burden. The model input signals and their fixed sensor and feature ordering are defined in Section 2.4.

The participant performed eight activities covering static postures, locomotion, directional turning, and transitional lower-body movements: Standing, Sitting, Walking, Stairs cycle, Turning (L/R), Sit-to-Stand cycle, Squatting, and Stooping. These activities are relevant to practical HAR applications, including rehabilitation and industrial safety. In particular, Squatting and Stooping involve coordinated lower-body and trunk movements. The Sit-to-Stand cycle is relevant to wearable-sensor-based assessment of postural transitions and fatigue [28].

Each activity was performed according to predefined guidelines to standardize the recording conditions. The detailed measurement methods, durations, repetition counts, and representative sensor signals are summarized in Table 1. A 1 min rest period was provided between consecutive recording files to minimize fatigue and maintain consistency across recordings. Standing and Sitting were maintained for approximately 1 min, Walking was performed on a treadmill at 1m/s, and Turning involved repeated 90° and 180° rotations in both directions; Squatting and Stooping followed predefined repetition counts. Compound activities were organized into predefined sequential cycles. The Stairs cycle consisted of stair ascent, a 180° left turn, stair descent, and a 180° right turn. The Sit-to-Stand cycle consisted of sitting down, stabilized sitting, standing up, and stabilized standing.

### 3.2. Annotation and Labeling

The synchronized continuous IMU recordings were annotated at the sample level according to the predefined activity start and end criteria summarized in Table 2. The eight activities were represented using 15 unique activity and transition labels. For concise references to activity labels and activity-label sequences, the abbreviated notation defined in Table 2 is used hereafter, whereas the experimental activities are described using their full names. These explicit start and end criteria were used to improve the consistency and reproducibility of sample-level annotation. They were particularly important for transitional labels whose phase boundaries can otherwise be difficult to define consistently.

The compound activities were annotated as continuous sequences of static and transitional labels. The Stairs cycle followed SR_U → TR_L → SR_D → TR_R. The Sit-to-Stand cycle followed ST → SI_D → SI → ST_U → ST. Squatting followed ST → SQ_D → SQ → SQ_U → ST, whereas Stooping followed ST → SP_D → SP → SP_U → ST. Independent Turning trials alternated between ST and the corresponding direction-specific TR_L or TR_R label. The TR_L and TR_R labels were shared between the turning phases embedded in the Stairs cycle and the independently recorded Turning trials.

**Figure 3:**
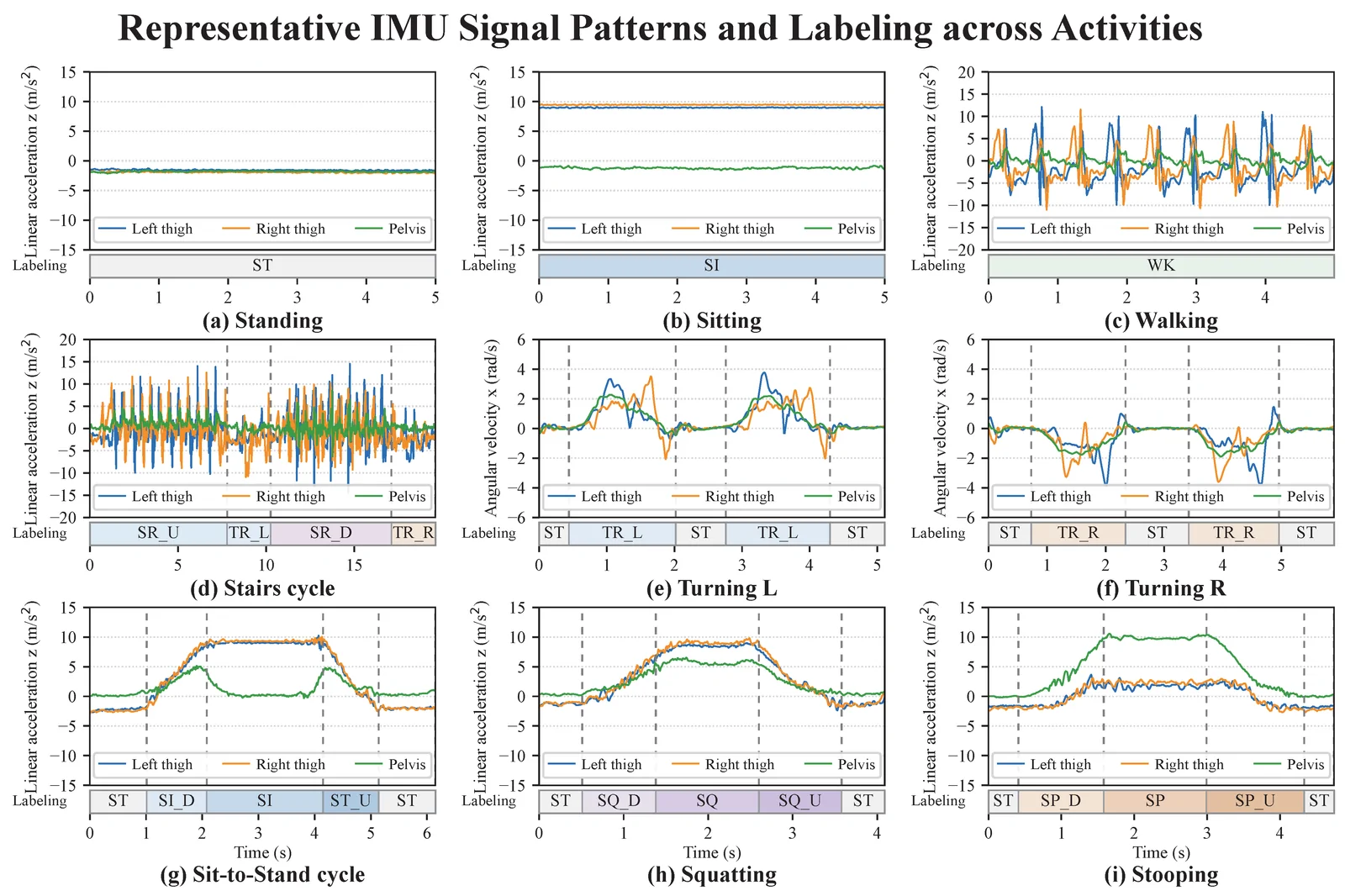
Representative IMU signal patterns and activity labeling for the eight activities, with Turning L and R shown separately: (**a**) Standing, (**b**) Sitting, (**c**) Walking, (**d**) Stairs cycle, (**e**) Turning L, (**f**) Turning R, (**g**) Sit-to-Stand cycle, (**h**) Squatting, and (**i**) Stooping. Raw *z*-axis linear accelerations from the pelvis and both thighs are shown for all activities except turning, for which the raw *x*-axis angular velocities are presented. The colored bars indicate the labels assigned to each activity phase, and the vertical dashed lines denote phase boundaries.

Figure 3 presents representative IMU signals together with the corresponding sample-level activity labels. Raw *z*-axis linear accelerations from the pelvis and both thighs are shown for Standing, Sitting, Walking, the Stairs cycle, the Sit-to-Stand cycle, Squatting, and Stooping. For Turning L/R, raw *x*-axis angular velocities are shown to visualize direction-dependent rotational patterns in the adopted sensor coordinate system. The colored bars indicate the assigned activity labels, while the vertical dashed lines denote the boundaries between successive activity phases. Static posture labels are generally associated with relatively stable signal intervals, locomotor labels exhibit periodic variations, and transitional labels are characterized by transient signal changes between successive postures. The displayed signal axes were selected solely for visualization, whereas all 18 features defined in Section 2.4 were retained as model inputs.

### 3.3. Data Preprocessing and Window Segmentation

Following sample-level annotation, the labeled recordings were segmented using a sliding window of length L=50 samples and stride S=25 samples. Sliding windows were generated independently within each recording and were not allowed to span recording boundaries. At a sampling frequency of 100Hz, the window length and stride corresponded to 0.5s and 0.25s, respectively, resulting in a 50% overlap between consecutive windows. For the *i*-th window within each recording, the starting sample index was defined as

(8) $$q_{i}=1+\left(i-1\right)S,$$

such that window *i* contained the samples from $q_{i}$ to $q_{i}+L-1$ within the corresponding recording.

Window labels were assigned by majority voting over the sample-level annotations. Let C denote the set of activity classes, and let $y_{t}$ denote the sample-level label at sample index *t*. The number of samples assigned to class *c* within window *i* was calculated as

(9) $$n_{i,c}=\sum_{k=0}^{L-1}I\left(y_{q_{i}+k}=c\right),\;c\in C,$$

where I(·) denotes the indicator function. The label of window *i* was then assigned as

(10) $${\tilde{y}}_{i}=\underset{c\in C}{\mathrm{arg max}}\;n_{i,c}.$$

To quantify the label consistency within each window, the majority-label proportion was defined as

(11) $$\rho_{i}=\frac{1}{L}\max_{c\in C}\;n_{i,c}.$$

Only windows satisfying $\rho_{i}\geq 0.60$ were retained, whereas windows with $\rho_{i}<0.60$ were discarded to remove ambiguous mixed-label windows near activity-transition boundaries. The threshold was selected through a sensitivity analysis of window retention, for which majority-label thresholds of 0.55, 0.60, 0.65, and 0.70 resulted in retention rates of 97.3%, 95.3%, 92.2%, and 89.9%, respectively. A threshold of 0.60 was selected to reduce ambiguous mixed-label windows while retaining most of the available data.

After purity filtering, the retained windows were partitioned using a two-stage group-wise file/session split into training, validation, and test sets with target proportions of 70%, 15%, and 15%, respectively. The split was adjusted as needed to ensure that all activity classes were represented in the training set while maintaining disjoint recording groups across the three subsets. The recording-file identifiers were preserved throughout preprocessing, and all windows originating from the same recording were assigned exclusively to one subset. This group-wise partitioning prevented temporally adjacent or overlapping windows from the same recording from appearing across different data splits.

After group-wise partitioning, the 18 IMU features were independently standardized using z-score normalization. A StandardScaler was fitted to the flattened samples of the training windows only, and the resulting statistics were applied without refitting to the validation and test windows. For the *j*-th feature, the normalized value was calculated as

(12) $${\hat{x}}_{t,j}=\frac{x_{t,j}-\mu_{j}^{\mathrm{train}}}{\sigma_{j}^{\mathrm{train}}},\;j=1,\ldots ,18,$$

where $x_{t,j}$ and ${\hat{x}}_{t,j}$ denote the original and normalized values of the *j*-th feature at sample index *t*, respectively, and $\mu_{j}^{\mathrm{train}}$ and $\sigma_{j}^{\mathrm{train}}$ denote the mean and standard deviation (std) calculated from the training-window samples for that feature. This procedure prevented information from the validation and test sets from influencing the preprocessing parameters. The normalized feature vector at sample index *t* was denoted by

(13) $${\hat{x}}_{t}={\left[{\hat{x}}_{t,1},\ldots ,{\hat{x}}_{t,18}\right]}^{T}\in R^{18}.$$

Using the previously defined window boundaries, the normalized input sequence for window *i* was constructed as

(14) $$X_{i}=\left[\begin{array}{c} {\hat{x}}_{q_{i}}^{T}\\ {\hat{x}}_{q_{i}+1}^{T}\\ \vdots \\ {\hat{x}}_{q_{i}+L-1}^{T} \end{array}\right]\in R^{L\times 18}.$$

Accordingly, each model input was a 50×18 matrix representing 50 temporal samples and 18 IMU features.

### 3.4. Training Procedure

All six model configurations—the Baseline and initial Lightweight variants of TCN, Transformer, and LSTM—were trained using the common 50×18 input representation. The Baseline and initial Lightweight models were independently initialized and trained from scratch under the same initial-training conditions. For each training run, the checkpoint achieving the highest validation macro F1-score was retained. For the Lightweight variants, the retained checkpoint was subsequently used as the starting point for the model-specific structured-pruning procedures described in Section 2.2.

Initial training was performed with a batch size of 64 for up to 80 epochs. Label smoothing [29] was applied to the loss with a factor of 0.1. Model parameters were optimized using AdamW [30], with an initial learning rate of $2\times 10^{-3}$ and a weight decay of $1\times 10^{-4}$. The learning rate was linearly warmed up from 20% of the initial learning rate during the first five epochs and was subsequently adjusted using cosine annealing. Gradient clipping with a maximum norm of 1.0 was applied, and early stopping with a patience of 14 epochs was used to reduce overfitting.

During initial training, light data augmentation was applied only to the standardized training windows to improve robustness to sensor noise and temporal variations; no augmentation was applied to the validation or test windows. Gaussian noise with σ=0.02 was applied with a probability of p=0.5, and random circular temporal shifts of up to ±5 frames were applied with a probability of p=0.3. The same augmentation procedure was applied to the Baseline and initial Lightweight models.

To mitigate class imbalance, a soft inverse-frequency WeightedRandomSampler with an exponent of α=0.3 was applied to the training data. The resulting class-dependent sampling multipliers were clipped to the interval [1/3,3], and training samples were drawn with replacement. No additional class weighting was applied to the loss.

During the pruning procedures, each candidate or progressively pruned model was briefly fine-tuned using a reduced learning rate of $5\times 10^{-4}$ to mitigate potential recognition-performance degradation. The fine-tuning duration and model-selection procedure were determined according to the corresponding model-specific pruning stage.

Within each model configuration, 10 independent training and evaluation runs were conducted using a fixed group-wise split and run-specific random seeds for initialization and training.

### 3.5. Implementation and Evaluation Platforms

The Baseline and Lightweight models were implemented in Python 3.10 using PyTorch 2.6. Model training was performed on a workstation equipped with an NVIDIA GeForce RTX 3060 GPU. Data preprocessing, dataset partitioning, and metric computation were implemented using scikit-learn 1.7.0, NumPy 1.26.1, and pandas 2.3.1, while result visualization was performed using NumPy 2.2.4 and Matplotlib 3.10.7.

Offline CPU evaluation was conducted on a Desktop platform equipped with an Intel Core i7-13700KF processor running Ubuntu 22.04. The offline latency measurements of the Baseline and Lightweight models were performed using single-thread FP32 inference with a batch size of one. The same Desktop platform, configured with ROS 2 Humble, was used for online streaming evaluation to assess model execution under the real-time IMU data-acquisition pipeline. The Quantized variants described in Section 2.3 were additionally validated on the Desktop platform using their final quantized artifacts before embedded evaluation.

Embedded online evaluation was conducted on a Raspberry Pi 4 Model B equipped with a quad-core ARM Cortex-A72 processor operating at 1.8GHz and 8GB of memory. The Raspberry Pi ran Ubuntu 22.04.5 LTS, ROS 2 Humble, Python 3.10, and PyTorch 2.9.1+cpu. The Baseline and Lightweight variants were evaluated using FP32 inference, whereas the Quantized variants were evaluated using their architecture-specific INT8 deployment configurations. The Quantized TCN was executed using ExecuTorch with the XNNPACK backend, whereas the Quantized Transformer and LSTM were executed using PyTorch with the QNNPACK backend.

Energy profiling during Raspberry Pi streaming was performed using PowerJoular [31], which was executed as a separate process to obtain process identifier (PID)-level software estimates of CPU power. The inference node recorded wall-clock timestamps and cumulative inference counts to temporally align the PowerJoular trace with the corresponding HAR streaming intervals. Because PowerJoular provides software-based estimates rather than direct measurements at the Raspberry Pi power input, the reported power and energy values are treated as estimated quantities.

### 3.6. Evaluation Protocol

We evaluated the Baseline and Lightweight models across four dimensions: classification performance, offline on-device efficiency, online on-device efficiency, and action-level trial success rates. The Quantized variants were additionally evaluated for classification performance on the Desktop platform and for online streaming efficiency, including energy profiling, on the Raspberry Pi.

- **Classification Performance**: Classification performance of the Baseline and Lightweight models was evaluated on the corresponding held-out group-wise test split using accuracy, loss, and macro-averaged precision, recall, and F1-score. Per-class F1-scores and row-normalized confusion matrices were additionally used to examine class-specific recognition characteristics. The classification metrics were reported as the mean ± std over the 10 independent training runs. For the quantization and deployment analyses, the final post-pruning Lightweight checkpoint obtained from the lightweighting procedure was used as the FP32 source model for each architecture. Each FP32 source checkpoint and its corresponding Quantized variant were evaluated on the same group-wise test windows using accuracy and macro-averaged F1-score to quantify changes after quantization.
- **Offline On-Device Efficiency**: Offline efficiency of the Baseline and Lightweight models was evaluated using parameters, on-disk FP32 model size, and CPU latency. Parameters were counted directly from each trained model, whereas on-disk FP32 model size was measured from the saved state-dictionary file. Measurements were performed on the Intel Core i7-13700KF Desktop platform using single-thread FP32 inference with a batch size of one and a sequence length of 50. Core latency was measured for the forward pass only using 50 warm-up runs followed by 500 timed runs. Inference latency was measured for the forward pass followed by argmax using 30 warm-up runs followed by 200 timed runs. Input standardization and tensor conversion were excluded from the timed Inference-latency interval. MACs per inference were estimated according to Section 2.5.
- **Online On-Device Efficiency**: Online efficiency was evaluated during continuous streaming inference on the Desktop and Raspberry Pi platforms. Desktop evaluation included the Baseline and Lightweight models, while Raspberry Pi evaluation additionally included the Quantized variants using the same streaming metrics and procedure. E2E latency was measured from the sensor-message timestamp to the prediction output, including message age and processing backlog. Pipeline latency was measured from the beginning of the inference callback to the prediction output, whereas Core latency measured only the model forward pass. Streaming throughput was evaluated using the Message Rate (Msg Rate) and Inference Rate (Infer Rate), representing the number of processed sensor messages and completed predictions per second, respectively. Latency and throughput were measured per activity and averaged across activities for each model–platform configuration. For the Raspberry Pi evaluation, software-estimated CPU power was additionally profiled during continuous 100Hz IMU streaming using PowerJoular. The PowerJoular trace and cumulative inference counts were temporally aligned with the corresponding HAR streaming intervals, and only the overlapping intervals were retained for energy analysis. Estimated total energy was obtained by trapezoidal integration of estimated power over the aligned intervals; average power and energy per inference were calculated by dividing total energy by aligned time and inference count, respectively.
- **Action-Level Trial Success Rate**: Action-level consistency was evaluated for the Baseline and Lightweight models from predictions published during each labeled action segment in the online pipeline, including transitional actions within compound sequences. A trial was considered successful when the intended action label accounted for at least 60% of the per-window predictions within that interval. For each model–platform condition, the success rate was calculated over six trials each for TR_L and TR_R and 10 trials each for SI_D, ST_U, SQ_D, SQ_U, SP_D, and SP_U.

This evaluation protocol enables a consistent comparison of recognition performance and deployment efficiency by jointly considering model-level computational workload, measured latency and streaming throughput, and software-estimated energy efficiency, while separately assessing the effects of INT8 quantization on classification performance and Raspberry Pi deployment behavior.

## 4. Results

The results are reported for the Baseline and Lightweight variants of TCN, Transformer, and LSTM, together with the additional quantization, computational complexity, and Raspberry Pi energy-efficiency evaluations defined in Section 3.6. The results are presented in the following order: overall classification performance, classification performance after quantization, offline on-device efficiency, computational complexity, online on-device efficiency, software-estimated energy efficiency on Raspberry Pi, class-level recognition characteristics, and action-level trial success rates.

### 4.1. Overall Classification Performance

As summarized in Table 3, among the Baseline models, TCN achieves the highest accuracy (95.8%), followed by LSTM (95.0%) and Transformer (94.0%); the ordering is consistent for F1-score as well (TCN 95.2%, LSTM 94.4%, Transformer 93.3%). For the Lightweight variants, the best accuracy is obtained by TCN at 93.6% (−2.2 percentage points (pp) vs. Baseline), while Transformer reaches 93.2% (−0.8 pp) and LSTM 93.1% (−1.9 pp). F1-score results show similar trends: TCN 93.0% (−2.2 pp), Transformer 92.7% (−0.6 pp), and LSTM 92.1% (−2.3 pp). Loss increases for all models (TCN 0.210→0.264; Transformer 0.240→0.277; LSTM 0.252→0.321). Overall, the Lightweight variants show accuracy reductions of 0.8–2.2 pp relative to their corresponding Baseline models; Transformer shows the smallest reduction, whereas TCN and LSTM incur larger reductions.

The precision and recall results provide further insight into the effects of lightweighting. For TCN, precision decreases from 95.0% to 94.0%, whereas recall decreases from 95.6% to 92.5%. For Transformer, precision increases slightly from 93.2% to 93.4%, while recall decreases from 93.7% to 92.3%. For LSTM, precision and recall decrease from 94.1% to 92.6% and from 94.8% to 91.8%, respectively. Thus, the performance reductions for TCN and LSTM are more pronounced in recall than in precision, motivating further examination through the subsequent per-class F1-score and confusion-matrix analyses.

### 4.2. Classification Performance After Quantization

Table 4 compares the final post-pruning Lightweight FP32 checkpoint used as the source model for quantization with its corresponding Quantized artifact for each architecture on the same group-wise test windows. For TCN, accuracy increases from 0.9404 to 0.9416 (+0.126 pp), while F1-score increases from 0.9351 to 0.9363 (+0.121 pp). For Transformer, accuracy decreases from 0.9366 to 0.9360 (−0.063 pp), while F1-score decreases from 0.9324 to 0.9318 (−0.063 pp). For LSTM, accuracy increases from 0.9372 to 0.9385 (+0.126 pp), while F1-score increases from 0.9274 to 0.9284 (+0.105 pp). Overall, the absolute changes in both accuracy and F1-score remain below 0.13 pp for all three architectures, indicating only minor classification-performance changes after INT8 quantization.

### 4.3. Offline On-Device Efficiency

**Figure 4:**
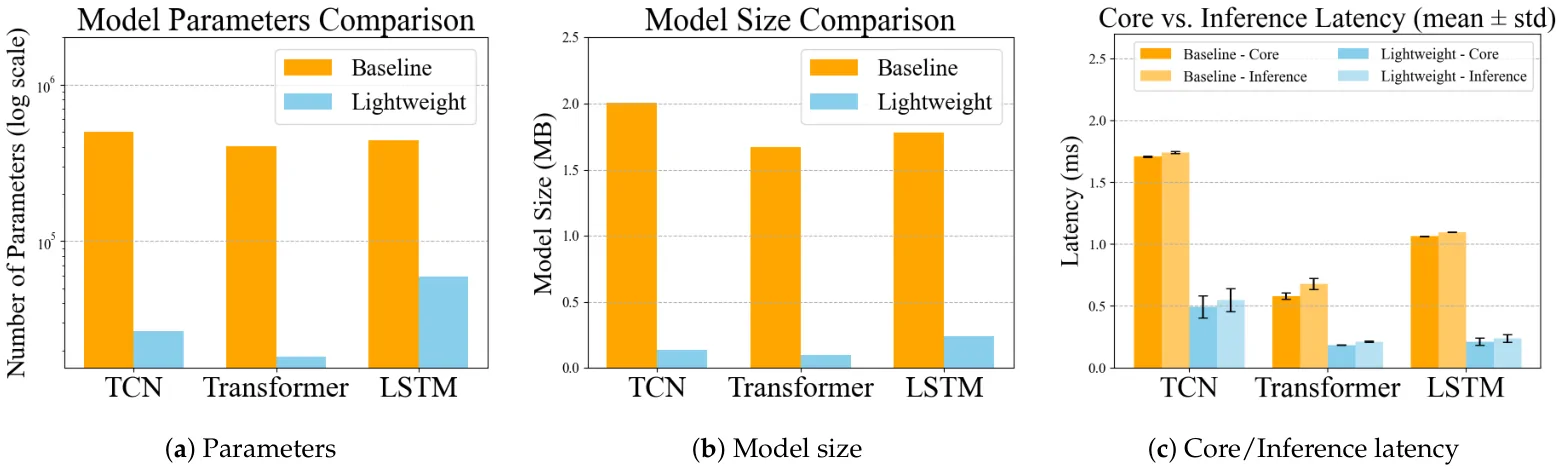
Offline on-device efficiency across Baseline and Lightweight variants: (**a**) parameters (log scale), (**b**) on-disk FP32 model size (MB), and (**c**) Core and Inference latency (mean ± std). Measurements were obtained on an Intel Core i7-13700KF using PyTorch 2.6 with single-thread FP32 inference, batch size =1, and input size 50×18. Core latency used 50 warm-up and 500 timed runs. Inference latency used 30 warm-up and 200 timed runs.

We compare Baseline and Lightweight variants in terms of parameters, on-disk FP32 model size, and Core/Inference CPU latency (Figure 4). For TCN, parameters are reduced by 94.7% (498.45K→26.60K) and model size by 93.5% (2.01→0.13MB). Core latency drops from 1.71 to 0.49ms (−71.3%), and Inference latency from 1.74 to 0.55ms (−68.4%). For Transformer, parameters are reduced by 95.5% (401.81K→18.26K) and model size by 94.0% (1.67→0.10MB). Core latency drops from 0.58 to 0.19ms (−67.2%), and Inference latency from 0.68 to 0.21ms (−69.1%). For LSTM, parameters are reduced by 86.6% (442.26K→59.09K) and model size by 86.5% (1.78→0.24MB), and latency also decreases substantially (Core 1.06→0.21ms, −80.2%; Inference 1.10→0.24ms, −78.2%). In short, lightweighting substantially reduces latency, model size, and parameters across all three architectures.

Across the three architectures, reductions in parameters and model size do not translate proportionally into reductions in CPU latency. Although TCN and Transformer achieve larger parameter reductions than LSTM, LSTM shows the largest relative reductions in both Core and Inference latency. Under the offline CPU benchmark, all three Lightweight variants achieve sub-millisecond Core and Inference latencies. Among them, Transformer has the fewest parameters, the smallest model size, and the lowest absolute latency, whereas LSTM achieves the largest relative latency reduction. These results indicate that the wall-clock efficiency gains obtained through lightweighting depend on the model architecture rather than solely on the degree of parameter reduction.

### 4.4. Computational Complexity

**Figure 5:**
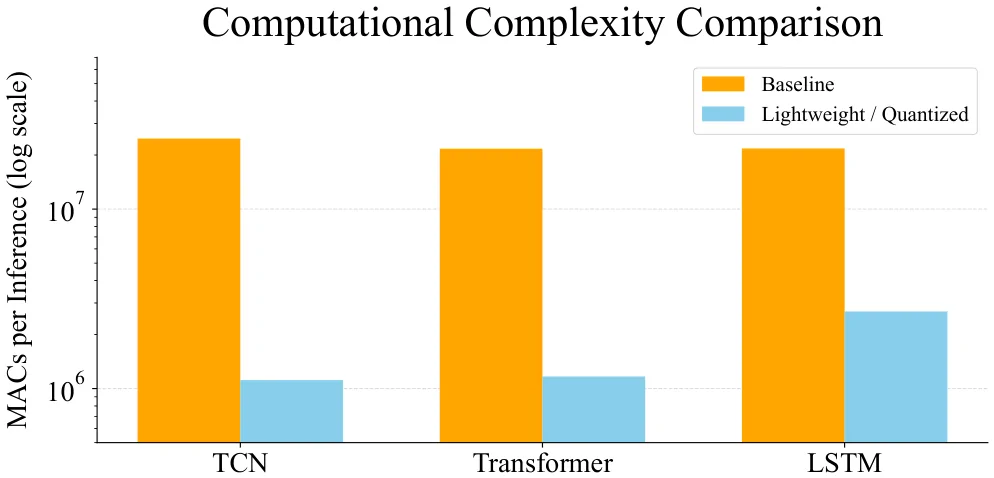
Model-level computational complexity of the evaluated TCN, Transformer, and LSTM architectures, expressed as multiply–accumulate operations (MACs) per inference for a single 50×18 input window with a batch size of one. The Lightweight and Quantized configurations are shown together because post-training INT8 quantization changes numerical precision and backend execution without changing the logical model architecture or MAC count.

Figure 5 compares the model-level computational complexity of the evaluated architectures using estimated MACs per inference for one 50×18 input window with a batch size of one, with the Lightweight and Quantized configurations shown together because they retain the same logical MAC counts. For TCN, lightweighting reduces the computational workload from 24.693 to 1.114 million MACs per inference, a 95.5% reduction. For Transformer, the computational workload decreases from 21.698 to 1.168 million MACs per inference (94.6%), while LSTM decreases from 21.762 to 2.689 million (87.6%). Across all three architectures, lightweighting substantially reduces model-level computational complexity, with TCN having the smallest MAC count among the Lightweight models, followed by Transformer and LSTM. However, these MAC reductions do not directly determine Raspberry Pi latency or throughput, indicating that target-platform performance also depends on architecture-specific execution characteristics and backend implementation.

### 4.5. Online On-Device Efficiency

Table 5 reports online on-device efficiency measured during streaming inference on Desktop and Raspberry Pi platforms; the values are averaged across the evaluated activities. On Desktop, all models sustain Msg and Infer Rates of approximately 100Hz under both Baseline and Lightweight settings. Lightweighting consistently reduces E2E, Pipeline, and Core latencies. For TCN, E2E latency decreases from 7.785 to 6.988ms, while Core latency decreases from 2.237 to 1.867ms. For Transformer, E2E latency decreases from 6.209 to 3.205ms, while Core latency decreases from 0.848 to 0.355ms. For LSTM, E2E latency decreases from 8.469 to 6.500ms, while Core latency decreases from 4.127 to 0.962ms. Overall, lightweighting improves responsiveness on Desktop while maintaining message and inference throughput at approximately 100Hz.

On Raspberry Pi, the Baseline models exhibit substantially larger latencies and lower throughput than on Desktop, indicating that the streaming pipeline becomes compute-limited under the embedded workload. Baseline TCN and LSTM show Msg Rates of 51.448 and 34.611Hz and Infer Rates of 22.289 and 14.124Hz, respectively. Baseline Transformer performs better than the other Baseline models, with an E2E latency of 16.745ms, a Core latency of 12.752ms, a Msg Rate of 80.097Hz, and an Infer Rate of 42.991Hz.

Lightweighting improves the Raspberry Pi results for all three architectures. Lightweight Transformer achieves the best streaming performance among the Lightweight models, reducing E2E latency to 9.381ms and Core latency to 4.656ms, while increasing the Msg Rate to 99.358Hz and the Infer Rate to 66.925Hz. Lightweight LSTM shows the largest relative improvement, reducing E2E latency from 59.599 to 17.782ms and Core latency from 55.469 to 13.690ms, while increasing the Infer Rate from 14.124 to 40.001Hz. Lightweight TCN shows more moderate improvements, with E2E latency decreasing from 37.373 to 30.120ms and Infer Rate increasing from 22.289 to 24.836Hz. The Lightweight Transformer recovers a near-target Msg Rate of 99.358Hz, but its Infer Rate remains at 66.925Hz, indicating that near-target message processing does not necessarily imply an equivalent completed-prediction throughput under embedded execution. These results show that lightweighting consistently improves Raspberry Pi streaming efficiency, although the magnitude of the improvement differs substantially across architectures.

The effects of subsequent INT8 quantization are architecture-dependent. Relative to the Lightweight FP32 variants, E2E/Core latency for TCN decreases from 30.120/25.986 to 9.066/4.224ms, while Infer Rate increases from 24.836 to 69.928Hz. In contrast, E2E/Core latency for Transformer increases from 9.381/4.656 to 13.067/7.655ms, while Infer Rate decreases from 66.925 to 46.279Hz. For LSTM, E2E/Core latency increases from 17.782/13.690 to 22.333/16.951ms, while Infer Rate decreases from 40.001 to 31.868Hz. Among all Raspberry Pi configurations, the Quantized TCN achieves the lowest E2E/Core latencies and the highest Infer Rate. The Quantized TCN and Transformer recover near-target Msg Rates of 99.814 and 99.791Hz, respectively, while the Quantized LSTM reaches 96.679Hz; however, no Raspberry Pi configuration reaches a 100Hz Infer Rate.

### 4.6. Software-Estimated Energy Efficiency on Raspberry Pi

Table 6 summarizes the software-estimated energy efficiency during Raspberry Pi streaming. The table additionally reports the accumulated aligned streaming time, estimated total energy, and aligned inference workload used to derive the power- and energy-efficiency metrics. Because the aligned streaming duration and inference workload differ across configurations, estimated energy per inference is used as the work-normalized comparison metric rather than estimated total energy alone. Average estimated CPU power remains near 3W for most configurations, ranging from 2.983 to 3.049W, whereas the Quantized TCN shows a higher value of 3.465W.

Lightweighting reduces estimated energy per inference from 137.101 to 121.533mJ for TCN (11.4%), from 70.554 to 45.282mJ for Transformer (35.8%), and from 216.170 to 76.330mJ for LSTM (64.7%). The effect of INT8 quantization is architecture-dependent: energy per inference further decreases for TCN from 121.533 to 49.933mJ (58.9%), whereas it increases for Transformer from 45.282 to 64.977mJ (43.5%) and for LSTM from 76.330 to 96.168mJ (26.0%).

### 4.7. Class-Level Recognition Characteristics

**Figure 6:**
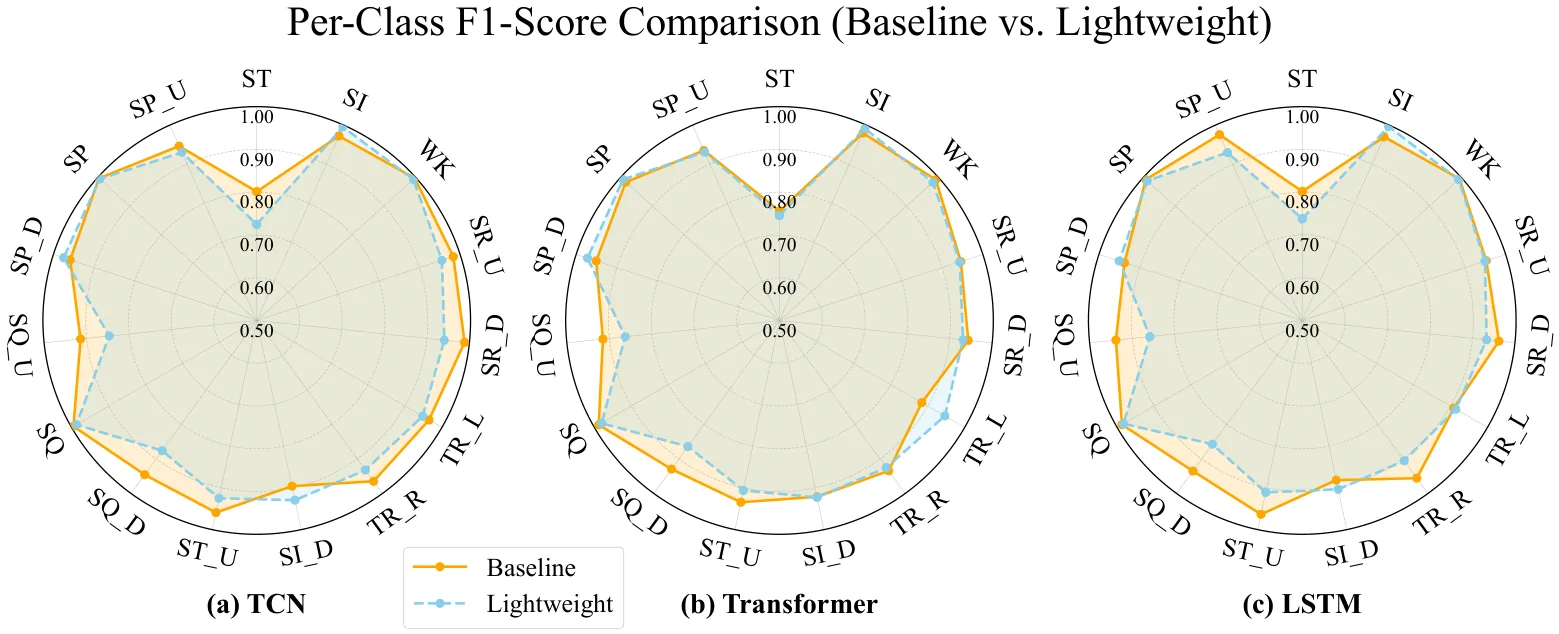
Radar chart comparison of the per-class F1-scores between the Baseline and Lightweight variants for (**a**) TCN, (**b**) Transformer, and (**c**) LSTM. Each radial axis corresponds to an activity class, and the radial distance indicates the mean F1-score over 10 independent runs. The radial range is restricted to 0.50–1.00 to highlight class-level performance differences between the two variants.

**Figure 7:**
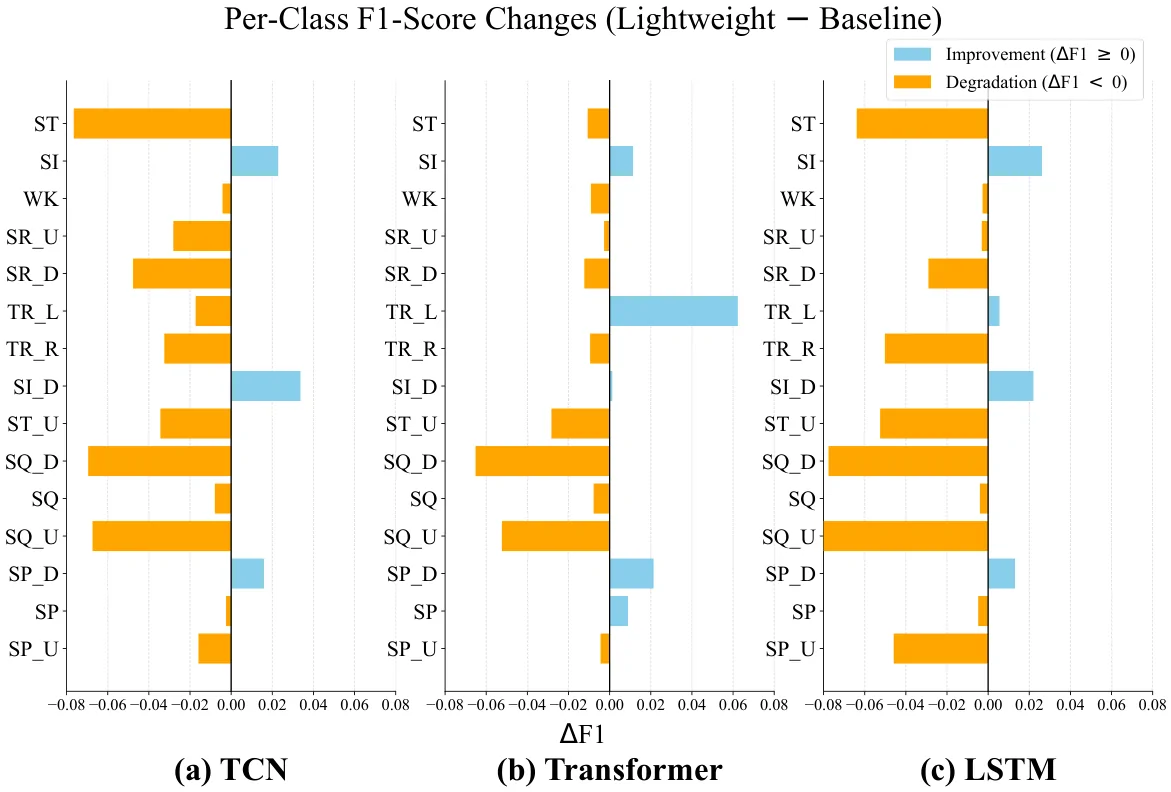
Per-class F1-score changes between the Lightweight and Baseline variants for (**a**) TCN, (**b**) Transformer, and (**c**) LSTM. The change is defined as $\Delta F1=F1_{\mathrm{Lightweight}}-F1_{\mathrm{Baseline}}$. Blue bars indicate improvement, whereas orange bars indicate degradation.

Figure 6 presents the per-class F1-scores of the Baseline and Lightweight variants, whereas Figure 7 shows the corresponding changes after lightweighting, defined as $\Delta F1=F1_{\mathrm{Lightweight}}-F1_{\mathrm{Baseline}}$. Across all three architectures, SI_D, SI, and SP_D improve, whereas the most consistent reductions occur for SQ_D and SQ_U. For TCN, the largest reductions are observed for ST (−0.0765), SQ_D (−0.0695), and SQ_U (−0.0674), while SI_D (+0.0337), SI (+0.0229), and SP_D (+0.0160) improve. For Transformer, TR_L shows the largest improvement (+0.0623), while SQ_D (−0.0652) and SQ_U (−0.0524) show the largest reductions. For LSTM, the largest reductions occur for SQ_U (−0.0800), SQ_D (−0.0776), and ST (−0.0639), whereas SI (+0.0262), SI_D (+0.0220), and SP_D (+0.0131) improve. The absolute changes for the maintained SQ phase and WK remain below 0.01 across all architectures, indicating that these classes are largely preserved after lightweighting.

**Figure 8:**
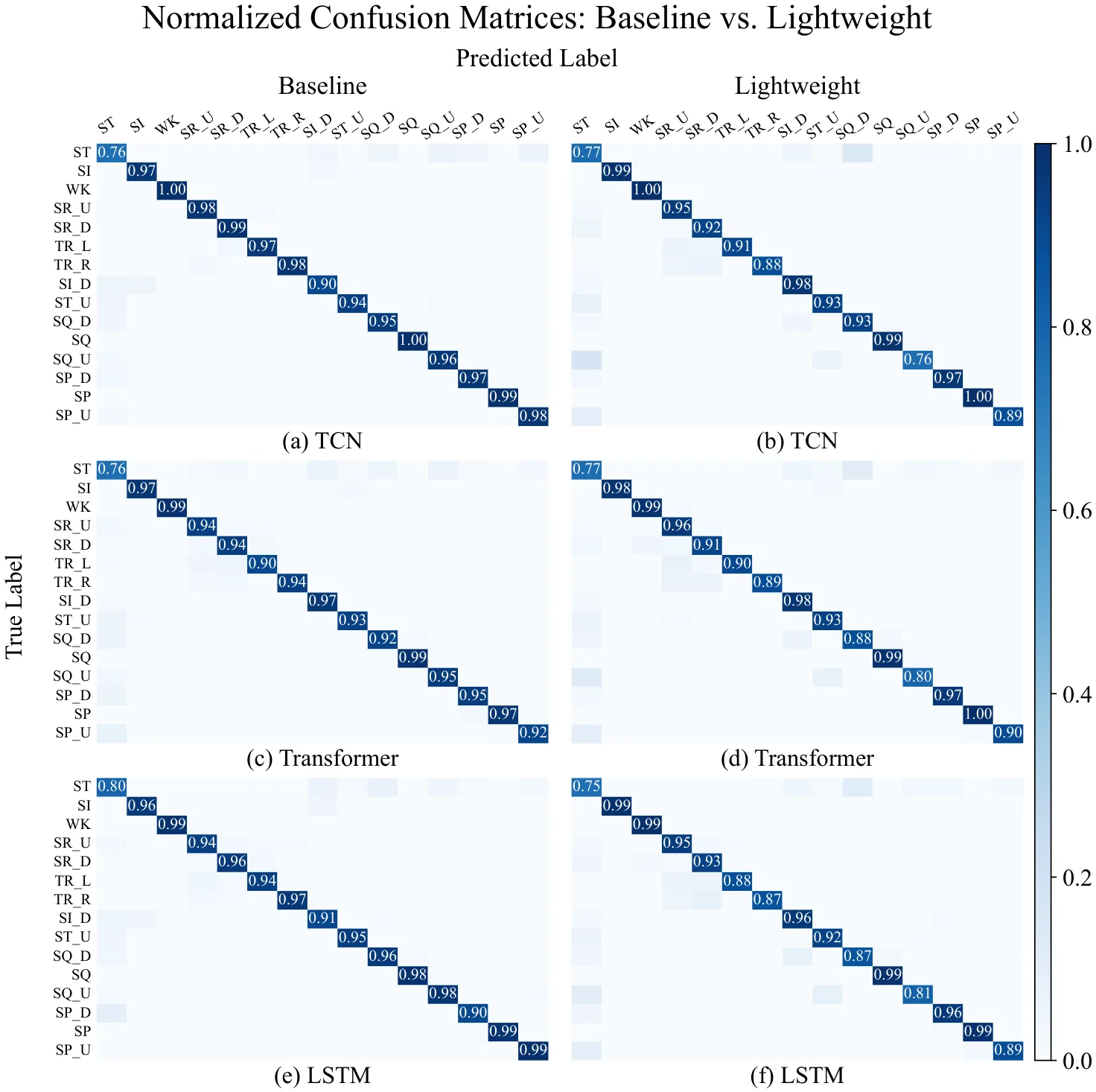
Normalized confusion matrices comparing the Baseline and Lightweight variants for the three evaluated sequence models. The left column presents the Baseline results and the right column presents the corresponding Lightweight results for (**a**,**b**) TCN, (**c**,**d**) Transformer, and (**e**,**f**) LSTM. Rows indicate the true activity labels and columns indicate the predicted activity labels. For each model variant, the confusion matrix was row-normalized within each independent run and subsequently averaged across 10 runs. Numerical annotations are displayed only for the main diagonal entries, which correspond to mean class-wise recall.

Figure 8 provides complementary information through normalized confusion matrices, whose diagonal values represent class recall. ST shows relatively low diagonal values for the Baseline TCN, Transformer, and LSTM models (0.76, 0.76, and 0.80) and remains challenging after lightweighting (0.77, 0.77, and 0.75). For the Lightweight models, the SQ_U diagonal values are 0.76, 0.80, and 0.81, while the SQ_D diagonal values are 0.93, 0.88, and 0.87 for TCN, Transformer, and LSTM, respectively. Among the Lightweight models, the dominant errors occur between adjacent phases: SQ_U is predicted as ST with proportions of 0.17, 0.12, and 0.10 for TCN, Transformer, and LSTM, respectively; ST is predicted as SQ_D with 0.14, 0.11, and 0.11; and SP_U is predicted as ST with 0.10, 0.10, and 0.09. In contrast, the maintained SQ phase and WK retain diagonal values of 0.99 and 0.99–1.00, respectively. Taken together, the results show that several of the largest remaining errors are concentrated around transitions into or out of ST, whereas maintained or periodic classes such as SQ and WK remain comparatively robust.

### 4.8. Action-Level Trial Success Rates

**Figure 9:**
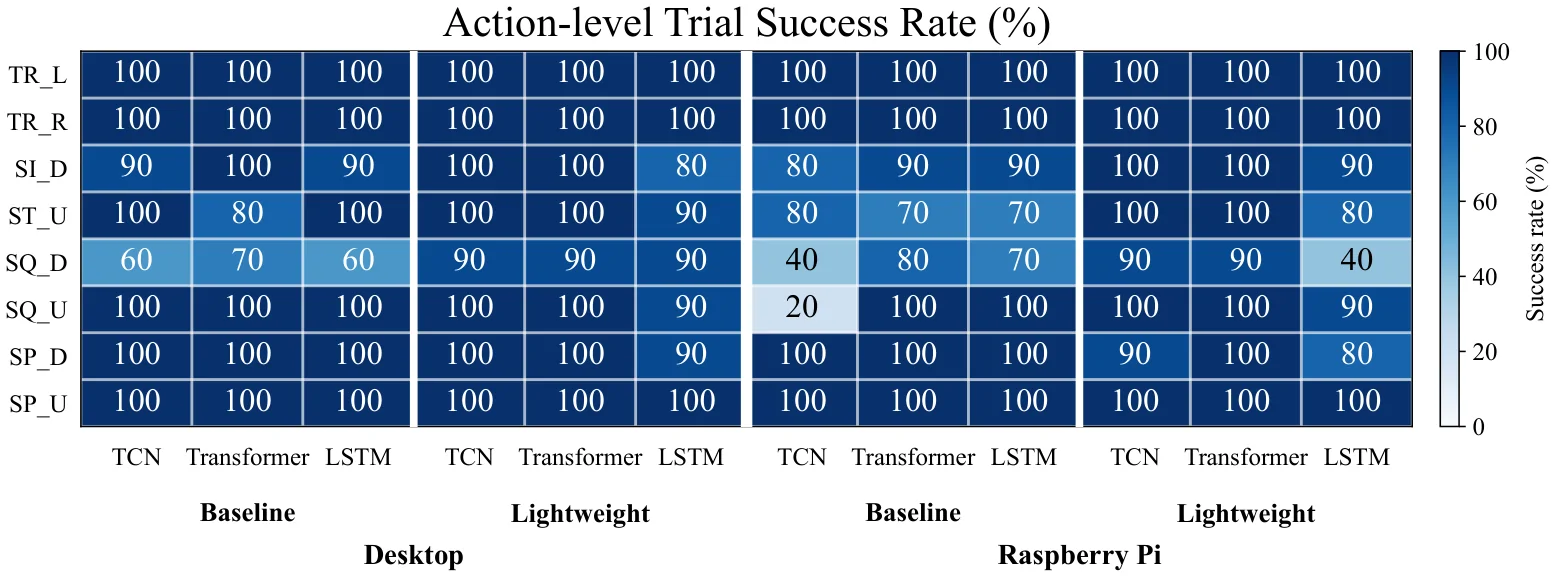
Action-level trial success rates across model variants and target platforms. A trial was considered successful when the intended action label accounted for at least 60% of the per-window predictions published during the corresponding action interval. Each cell represents the percentage of successful trials, calculated over six trials each for TR_L and TR_R and 10 trials each for SI_D, ST_U, SQ_D, SQ_U, SP_D, and SP_U under the corresponding model–platform condition.

Figure 9 shows the action-level trial success rates obtained using the protocol described in Section 3.6. This metric evaluates trial-level prediction consistency rather than window-level classification performance.

On Desktop, TR_L, TR_R, and SP_U achieve a success rate of 100% across all model configurations. The most notable weakness among the Baseline models occurs for SQ_D, with success rates of 60%, 70%, and 60% for TCN, Transformer, and LSTM, respectively. The corresponding Lightweight models improve these rates to 90% for all three architectures. Lightweight TCN and Transformer otherwise maintain success rates of 100% for most evaluated actions, whereas Lightweight LSTM shows slightly lower rates for SI_D (80%), ST_U (90%), SP_D (90%), and SQ_U (90%).

On Raspberry Pi, the trial success rates become more model- and action-dependent. Baseline TCN shows the largest degradation for the Squatting-related actions, with SQ_D and SQ_U success rates of 40% and 20%, respectively. In comparison, Baseline Transformer achieves 80% and 100%, while Baseline LSTM achieves 70% and 100% for the same actions. Lightweight Transformer provides the most consistent Raspberry Pi results, achieving 100% for all evaluated actions except SQ_D (90%). Lightweight TCN also improves SQ_D from 40% to 90% and SQ_U from 20% to 100%. In contrast, Lightweight LSTM shows mixed behavior: ST_U improves from 70% to 80%, whereas SP_D, SQ_D, and SQ_U decrease from 100%, 70%, and 100% to 80%, 40%, and 90%, respectively. Overall, lightweighting improves action-level consistency most clearly for Transformer and for the Squatting-related actions of TCN, but the effect is not uniform across architectures and actions.

## 5. Discussion

Our results reveal architecture-dependent trade-offs between recognition performance and on-device efficiency in the proposed L-HAR framework. Across all three architectures, lightweighting substantially reduces model footprint and offline CPU latency while limiting the accuracy reductions to 0.8–2.2 pp. Among the Lightweight variants, TCN retains the highest classification performance, Transformer combines the smallest accuracy and F1-score reductions with the smallest footprint and lowest absolute Core/Inference latency, and LSTM achieves the largest relative latency reduction. The different relationships between parameter reduction and latency improvement indicate that wall-clock efficiency depends on architecture-specific execution characteristics and the runtime environment rather than on parameter count alone. Accordingly, model selection for on-device HAR should consider absolute target-platform performance in addition to compression ratios.

Class-level changes are concentrated in specific transition phases. Although SI_D, SI, and SP_D improve across all architectures, degradation is most consistent for SQ_D and SQ_U, with several errors involving transitions into or out of ST. In contrast, the maintained SQ phase and the periodic WK class remain comparatively robust after lightweighting. Because the adopted 0.5 s windows can contain temporally adjacent motion characteristics, short transition phases near ST and SQ may remain more sensitive to boundary ambiguity even after purity filtering. This suggests that the Lightweight models preserve stable or repetitive motion patterns more readily than some short-duration transitions.

The online results show that offline efficiency does not directly determine streaming feasibility. On Desktop, all Baseline and Lightweight configurations sustain Msg Rate and Infer Rate of approximately 100Hz, and lightweighting mainly reduces E2E, Pipeline, and Core latencies. On Raspberry Pi, however, all evaluated configurations become more compute-limited, and none reaches a 100Hz Infer Rate. Among the Lightweight variants, Transformer provides the best streaming performance, with an E2E latency of 9.381ms and an Infer Rate of 66.925Hz, while recovering a near-target Msg Rate of 99.358Hz. The difference between Msg Rate and Infer Rate shows that processing incoming messages does not necessarily correspond to completing predictions at the same rate. The Lightweight Transformer results also directly illustrate the discrepancy between offline and online measurements: in the offline CPU benchmark on Desktop, it achieves a Core latency of 0.19ms, whereas during online streaming, its Core latency increases to 0.355ms on Desktop and 4.656ms on Raspberry Pi. On Raspberry Pi, its Pipeline latency is 5.890ms, and the higher E2E latency reflects additional pre-callback delay, including message age and processing backlog, that is not included in the Pipeline measurement. Because these system-level effects are not captured by the isolated offline benchmark, target-platform latency and sustained completed-prediction throughput should be verified through direct online measurements of E2E latency, Msg Rate, and Infer Rate on the deployment target.

The quantization results further demonstrate that INT8 quantization does not necessarily translate into uniform target-platform speedups. Across all three architectures, the absolute changes in accuracy and F1-score remain below 0.13 pp after quantization. On Raspberry Pi, the Quantized TCN substantially improves over its Lightweight FP32 counterpart, achieving an E2E latency of 9.066ms, a Core latency of 4.224ms, and an Infer Rate of 69.928Hz, whereas the Quantized Transformer and LSTM configurations show higher E2E and Core latencies and lower Infer Rates than their corresponding Lightweight FP32 variants. These results indicate that the practical benefit of the architecture-specific INT8 deployment configurations is model- and backend-dependent and should be verified directly on the target platform rather than inferred solely from numerical precision.

The energy-profiling results provide an additional perspective on target-platform deployment efficiency. Lightweighting reduces the estimated energy per inference by 11.4%, 35.8%, and 64.7% for TCN, Transformer, and LSTM, respectively. Among the architecture-specific INT8 deployment configurations, the estimated energy per inference decreases for TCN by 58.9% to 49.933mJ relative to its Lightweight FP32 variant, whereas the Quantized Transformer and LSTM configurations show increases of 43.5% and 26.0%, respectively. Together with the near-3W average software-estimated CPU power for most configurations, these results support evaluating energy efficiency using work-normalized energy per inference rather than total energy, average power, model size, or numerical precision alone.

The computational-complexity analysis provides a complementary model-level perspective on these deployment results. Lightweighting reduces the estimated MAC workload by 95.5%, 94.6%, and 87.6% for TCN, Transformer, and LSTM, respectively. However, these reductions do not translate proportionally into target-platform latency or energy efficiency, and the Quantized variants retain the same logical MAC counts as their corresponding Lightweight models despite differing Raspberry Pi deployment behavior. Thus, logical computational complexity should be considered together with direct target-platform latency, throughput, and energy measurements.

Action-level performance remains generally high on Desktop but becomes more architecture- and action-dependent on Raspberry Pi. Among the Lightweight variants, Transformer provides the most consistent results, TCN improves the Squatting-related transitions, and LSTM shows mixed changes across actions. The greater variation on Raspberry Pi may reflect the combined effects of class ambiguity and reduced completed-prediction throughput, particularly for short transition intervals, although their individual contributions were not separately quantified. Thus, improvements in offline latency or streaming throughput do not guarantee uniform gains in action-level prediction consistency.

The primary limitation is the single-participant dataset, which prevents the reported classification scores and class-specific error patterns from being interpreted as population-level performance. Nevertheless, the controlled setting enables the effects of model architecture, lightweighting strategy, and target platform to be examined under a fixed sensing configuration and runtime pipeline. The trial-level results should also be interpreted within the adopted 60% success criterion and controlled action intervals. In addition, missing or dropped IMU samples were not explicitly handled during streaming, and the energy analysis relied on software-estimated CPU power rather than direct board-level electrical measurements. Future work should extend the evaluation to multiple participants and hardware platforms, include complementary validation using standardized public HAR datasets and their established evaluation protocols, incorporate explicit missing-data handling and direct board-level electrical power measurements, and improve the recognition of short Squatting-related transitions.

## 6. Conclusions

In this work, we conducted a controlled, deployment-oriented comparison of Baseline and Lightweight TCN, Transformer, and LSTM models within the proposed L-HAR framework using three IMUs placed on the pelvis and both thighs. The framework jointly evaluated classification performance, model footprint, model-level computational complexity, offline CPU latency on Desktop, online streaming behavior on Desktop and Raspberry Pi, software-estimated energy efficiency on Raspberry Pi, and action-level prediction consistency on both platforms. The models were compared under common sensing, input, preprocessing, initial-training, and evaluation conditions, with architecture-specific lightweighting procedures applied to the Lightweight variants. Post-training INT8 quantization was additionally evaluated for the Lightweight models.

The combined evaluations showed that lightweighting substantially reduced model footprint and offline CPU latency while limiting accuracy reductions to 0.8–2.2 pp, with all Lightweight variants achieving sub-millisecond offline Core and Inference latencies. Among them, TCN retained the highest classification performance, Transformer combined the smallest accuracy and F1-score reductions with the smallest footprint and lowest absolute offline Core/Inference latencies, and LSTM achieved the largest relative offline latency reduction. After INT8 quantization, the absolute changes in accuracy and F1-score remained below 0.13 pp for all three architectures. All Baseline and Lightweight configurations sustained approximately 100Hz Msg Rate and Infer Rate on Desktop, whereas none of the evaluated Raspberry Pi configurations reached a 100Hz Infer Rate. Among the Lightweight variants, Transformer provided the best Raspberry Pi streaming performance, with an E2E latency of 9.381ms, a Msg Rate of 99.358Hz, and an Infer Rate of 66.925Hz. On Raspberry Pi, the Quantized TCN achieved the best overall streaming performance (9.066ms E2E; 4.224ms Core; 69.928Hz Infer Rate), whereas the Quantized Transformer and LSTM configurations showed higher E2E/Core latencies and lower Infer Rates than their corresponding Lightweight FP32 variants. Energy profiling further showed that lightweighting reduced the software-estimated energy per inference for all three architectures. Regarding energy efficiency, the Quantized TCN configuration yielded a software-estimated energy per inference of 49.933mJ, lower than its Lightweight FP32 counterpart, whereas the Quantized Transformer and LSTM configurations yielded higher values than their corresponding Lightweight FP32 variants. The computational-complexity analysis further showed that lightweighting reduced the estimated MAC workload by 95.5%, 94.6%, and 87.6% for TCN, Transformer, and LSTM, respectively. Class- and action-level analyses further showed that lightweighting effects were not uniform, with degradation concentrated in SQ_D, SQ_U, and ST-related transitions and trial-level changes varying across architectures and platforms. The discrepancy between offline and online performance also demonstrates that favorable offline Core latency does not necessarily translate into equivalent target-platform streaming performance, because isolated offline benchmarks do not capture message age, processing backlog, or the difference between Msg Rate and Infer Rate.

Overall, these results demonstrate the value of unified, controlled target-platform evaluation and show that practical on-device HAR models should be selected based on recognition performance, model footprint, computational workload, streaming latency and throughput, energy efficiency, and class- and action-level consistency rather than parameter reduction, logical operation count, numerical precision, or offline latency alone.

## Figures and Tables

**Table 1:** Human activity data collection guidelines used in the proposed L-HAR framework. A 1 min rest period was provided between consecutive recording files to minimize fatigue and maintain consistency across recordings. The “Main Sensor Signals” column highlights representative signals for interpreting activity-specific motion patterns. All 18 IMU features were retained as model inputs. Here, acc and gyro denote linear acceleration and angular velocity, respectively.

Activity	Measurement Method	Duration/Repetitions	Main Sensor Signals
Standing	Facing forward, maintaining upright posture	60 s × 3 files (≈3 min)	Pelvis acc, both thighs’ acc
Sitting	Chair without backrest, maintaining upright posture	60 s × 3 files (≈3 min)	Pelvis acc, both thighs’ acc
Walking	Treadmill walking at a fixed speed of 1.0 m/s	60 s × 3 files (≈3 min)	Both thighs’ gyro/acc
Stairs cycle	Cycle = stair ascent + 180° left turn + stair descent + 180° right turn	1 file = 5 cycles × 4 files = total 20 cycles (≈6 min)	Both thighs’ gyro/acc
Turning (L/R)	90° left/right turn + 180° left/right turn 1st and 2nd files: left/3rd and 4th files: right	90° (15 times) + 180° (10 times) × 4 files (≈4 min)	Pelvis gyro, both thighs’ gyro
Sit-to-Stand cycle	Cycle = sit down + stabilized sitting + stand up + stabilized standing	1 file = 20 cycles × 3 files = total 60 cycles (≈5 min)	Pelvis acc/gyro, both thighs’ acc/gyro
Squatting	Maintain the thighs approximately parallel to the floor	20 reps × 4 files (total ≈ 5 min)	Pelvis acc/gyro, both thighs’ acc/gyro
Stooping	Maintain the trunk approximately parallel to the floor	20 reps × 4 files (total ≈ 5 min)	Pelvis acc/gyro, both thighs’ acc/gyro

**Table 2:** Activity labels, abbreviations, and sample-level start/end annotation criteria used in the proposed L-HAR framework.

Activity	Label (Abbreviation)	Label Start/End Criteria
Standing	STAND (ST)	Start: Upright standing posture is maintained after completion of a preceding movement. End: Initiation of walking, sitting, turning, squatting, stooping, or stair movement.
Sitting	SIT (SI)	Start: Seated posture is stabilized after completion of SI_D. End: Initiation of ST_U or another movement.
Walking	WALK (WK)	Start: First continuous gait cycle after movement initiation. End: Termination of continuous gait before standing or another activity.
Stairs cycle	STAIR_UP (SR_U), STAIR_DOWN (SR_D)	SR_U: From initiation of upward stepping to reaching the upper landing. SR_D: From initiation of downward stepping to reaching the lower landing.
Turning (L/R)	TURN_L (TR_L), TURN_R (TR_R)	Start: Initiation of leftward or rightward body rotation. End: Completion of the intended 90° or 180° rotation and posture stabilization.
Sit-to-Stand cycle	SIT_DOWN (SI_D), SIT (SI), STAND_UP (ST_U)	SI_D: From initiation of trunk/hip flexion to stabilized sitting posture. SI: Maintained seated posture between SI_D and ST_U. ST_U: From initiation of rising to stabilized upright standing posture.
Squatting	SQUAT_DOWN (SQ_D), SQUAT (SQ), SQUAT_UP (SQ_U)	SQ_D: From initiation of lower-limb flexion to the target squat posture. SQ: Maintained target posture with the thighs approximately parallel to the floor. SQ_U: From initiation of extension to recovery of upright standing posture.
Stooping	STOOP_DOWN (SP_D), STOOP (SP), STOOP_UP (SP_U)	SP_D: From initiation of trunk flexion to the target stooped posture. SP: Maintained target posture with the trunk approximately parallel to the floor. SP_U: From initiation of trunk extension to recovery of upright standing posture.

Note: The abbreviations in parentheses are used throughout the manuscript, figures, and tables for compact references to activity labels and activity-label sequences. The original class names are retained in the sample-level annotations, model-training pipeline, and raw model outputs.

**Table 3:** Classification performance of the Baseline and Lightweight variants on their corresponding held-out group-wise test splits (mean ± standard deviation over 10 independent runs). All models use the same preprocessing procedure, initial-training conditions, and evaluation protocol.

Stage	Model	Accuracy	Precision	Recall	F1-Score	Loss
Baseline	TCN	0.958 ± 0.004	0.950 ± 0.005	0.956 ± 0.004	0.952 ± 0.004	0.210 ± 0.017
	Transformer	0.940 ± 0.009	0.932 ± 0.009	0.937 ± 0.009	0.933 ± 0.009	0.240 ± 0.023
	LSTM	0.950 ± 0.008	0.941 ± 0.009	0.948 ± 0.008	0.944 ± 0.009	0.252 ± 0.027
Lightweight	TCN	0.936 ± 0.002	0.940 ± 0.002	0.925 ± 0.004	0.930 ± 0.003	0.264 ± 0.009
	Transformer	0.932 ± 0.004	0.934 ± 0.005	0.923 ± 0.006	0.927 ± 0.006	0.277 ± 0.018
	LSTM	0.931 ± 0.005	0.926 ± 0.006	0.918 ± 0.006	0.921 ± 0.006	0.321 ± 0.013

**Table 4:** Classification performance of the Lightweight FP32 checkpoints and their corresponding Quantized variants on the same group-wise test windows.

Model	FP32 Accuracy	INT8 Accuracy	Δ Accuracy (pp)	FP32 F1-Score	INT8 F1-Score	Δ F1-Score (pp)
TCN	0.9404	0.9416	+0.126	0.9351	0.9363	+0.121
Transformer	0.9366	0.9360	−0.063	0.9324	0.9318	−0.063
LSTM	0.9372	0.9385	+0.126	0.9274	0.9284	+0.105

Note: Δ denotes Quantized minus FP32 performance in percentage points (pp) and was calculated using the unrounded metric values.

**Table 5:** Online on-device efficiency during streaming inference on Desktop and Raspberry Pi, with values averaged across the evaluated activities. E2E includes message age and processing backlog from the sensor-message timestamp to the prediction output; Pipeline measures latency from callback start to prediction output; Core measures the forward pass only. Throughput is reported as Msg Rate and Infer Rate. The Quantized variants were evaluated only on Raspberry Pi.

Platform	Stage	Model	End-to-End (ms)	Pipeline (ms)	Core (ms)	Msg Rate (Hz)	Infer Rate (Hz)
Desktop	Baseline	TCN	7.785	2.323	2.237	100.001	100.096
		Transformer	6.209	0.933	0.848	100.002	100.088
		LSTM	8.469	4.209	4.127	100.000	99.730
	Lightweight	TCN	6.988	1.949	1.867	100.001	100.092
		Transformer	3.205	0.436	0.355	100.002	100.089
		LSTM	6.500	1.045	0.962	100.002	100.100
Raspberry Pi	Baseline	TCN	37.373	33.278	32.041	51.448	22.289
		Transformer	16.745	14.003	12.752	80.097	42.991
		LSTM	59.599	56.763	55.469	34.611	14.124
	Lightweight	TCN	30.120	27.258	25.986	74.719	24.836
		Transformer	9.381	5.890	4.656	99.358	66.925
		LSTM	17.782	14.946	13.690	84.905	40.001
	Quantized	TCN	9.066	5.591	4.224	99.814	69.928
		Transformer	13.067	8.801	7.655	99.791	46.279
		LSTM	22.333	18.197	16.951	96.679	31.868

**Table 6:** Software-estimated energy efficiency during continuous Raspberry Pi streaming. Aligned time, estimated total energy, and aligned inferences were accumulated over the HAR–PowerJoular overlapping intervals. Total energy was obtained by trapezoidal integration of the PowerJoular-estimated CPU power over the aligned intervals. The reported power and energy values are software-based estimates rather than direct board-level electrical measurements. Aligned inference values are rounded to the nearest whole inference for presentation, whereas the energy-per-inference values were calculated using the unrounded aligned inference workload.

Stage	Model	Aligned Time (s)	Est. Total Energy (J)	Aligned Inferences	Avg. Est. Power (W)	Est. Energy/Inf. (mJ)
Baseline	TCN	325.274	991.311	7231	3.048	137.101
	Transformer	302.563	912.128	12,928	3.015	70.554
	LSTM	350.568	1068.746	4944	3.049	216.170
Lightweight	TCN	309.958	933.036	7677	3.010	121.533
	Transformer	296.370	891.537	19,688	3.008	45.282
	LSTM	291.800	885.192	11,597	3.034	76.330
Quantized	TCN	269.015	932.164	18,668	3.465	49.933
	Transformer	291.309	869.022	13,374	2.983	64.977
	LSTM	292.465	888.176	9236	3.037	96.168

## Data Availability

The original data presented in the study are openly available in Zenodo at https://doi.org/10.5281/zenodo.21712401. The repository contains de-identified raw CSV files and labeled CSV files used for model training and evaluation.
